# NFL and GFAP in (pre)symptomatic RVCL-S carriers: a monogenic cerebral small vessel disease

**DOI:** 10.1007/s00415-024-12292-6

**Published:** 2024-04-06

**Authors:** Annelise E. Wilms, I. de Boer, N. Pelzer, S. G. J. G. in’t Veld, H. A. M. Middelkoop, C. E. Teunissen, G. M. Terwindt

**Affiliations:** 1 Present Address: Department of Neurology, Leiden University Medical Center, PO Box 9600, 2300RC Leiden, The Netherlands; 2 Neurochemistry Laboratory, Department of Clinical Chemistry, Amsterdam University Medical Centers, Vrije Universiteit Amsterdam, Amsterdam, The Netherlands; 3 Institute of Psychology, Health, Medical and Neuropsychology Unit, Leiden University, Leiden, The Netherlands

**Keywords:** Retinal vasculopathy with cerebral leukoencephalopathy and systemic manifestations (RVCL-S), Cerebral small vessel disease (SVD), Neuroinflammation, Neurofilament light chain (NfL), Glial fibrillary acidic protein (GFAP)

## Abstract

**Background:**

Neurofilament light chain (NfL) and glial fibrillary acidic protein (GFAP) have emerged as biomarkers for cerebral small vessel disease (SVD). We investigated their role in a hereditary SVD model, retinal vasculopathy with cerebral leukoencephalopathy and systemic manifestations (RVCL-S).

**Methods:**

NfL and GFAP levels of 17 pre-symptomatic, 22 symptomatic RVCL-S mutation carriers and 69 controls were measured using a Simoa assay. We assessed the association of serum and cerebrospinal fluid (CSF) levels of NfL and GFAP with RVCL-S symptomatology and neuropsychological functioning.

**Results:**

Serum and CSF NfL levels were higher in symptomatic RVCL-S compared to controls ≥ 45 years (33.5 pg/mL vs. 9.2 pg/mL, p < 0.01; 8.5*10^2^ pg/mL vs. 3.9*10^2^ pg/mL, p < 0.01, respectively). Serum NfL levels were higher in symptomatic RVCL-S than pre-symptomatic carriers (33.5 pg/mL vs. 5.9 pg/mL, p = 0.02). Pre-symptomatic RVCL-S carriers had increased CSF NfL levels compared to controls < 45 years (5.2*10^2^ pg/mL vs. 1.9*10^2^ pg/mL, p < 0.01). No differences were found in GFAP levels across groups, but in RVCL-S carriers higher serum levels of both NfL and GFAP were linked to poorer global cognitive functioning (β[95%CI] = − 2.86 [− 5.58 to − 0.13], p = 0.04 and β[95%CI] =  − 6.85 [− 11.54 to − 2.15], p = 0.01, respectively) and prolonged psychomotor test times (β[95%CI] = 6.71 [0.78–12.65], p = 0.03 and β[95%CI] = 13.84 [3.09–24.60], p = 0.01).

**Discussion:**

Higher levels of serum NfL and GFAP are associated with worse cognitive functioning in RVCL-S carriers and may serve as marker for disease progression. CSF NfL levels may serve as early marker as pre-symptomatic RVCL-S patients already show differences compared to young controls.

## Introduction

Retinal vasculopathy with cerebral leukoencephalopathy and systemic manifestations (RVCL-S) is an autosomal dominant vasculopathy caused by C-terminal truncating mutations in the *TREX1* gene [1–3]. The main features of RVCL-S are progressive neurological manifestations of focal and diffuse brain dysfunction and vascular retinopathy. Neuroimaging demonstrates white matter hyperintensities and intracerebral mass lesions (with gadolinium enhancement and/or diffusion restriction) [4, 5]. Less well described are the systemic manifestations such as liver and kidney dysfunction, hypertension, Raynaud’s phenomenon, hypothyroidism and anemia [5, 6]. It is unclear how the truncating *TREX1* mutations lead to vasculopathy, but it is suggested that endothelial dysfunction plays an important role [7–9]. RVCL-S serves as a monogenic model for cerebral small vessel disease (SVD) [1].

Neurofilament light-chain protein (NfL) and glial fibrillary acidic protein (GFAP) levels in serum and cerebrospinal fluid (CSF) have emerged as biomarkers for neurological diseases [10–13]. NfL is a marker of neuroaxonal damage [14]. In cases of sporadic SVD and the hereditary SVD variant CADASIL (cerebral autosomal dominant arteriopathy with subcortical infarcts and leukoencephalopathy) cognitive decline and disease progression have been linked to serum NfL levels [15–17]. GFAP is upregulated in reactive astrocytes and is therefore considered a marker for astrogliosis [18]. In Alzheimer's disease and other neurodegenerative dementias, elevated levels of GFAP are present in both serum and CSF, and associated with reduced cognitive performance [19–21].

As RVCL-S is a monogenic SVD it offers the opportunity to study the transition from the pre-symptomatic to the symptomatic phase of cerebral SVD. Early diagnostic testing and monitoring of disease activity remains a challenge in sporadic SVD, for which simple and efficient screening tools are needed. Identifying biomarkers of neuronal injury and reactive astrogliosis in RVCL-S would further support the hypothesis that SVD and neurodegenerative pathophysiology may be closely linked, perhaps through responses that drive neuroinflammation. Additionally, finding a serum biomarker offers numerous benefits over more challenging-to-acquire biomarkers such as high-field MRI markers or extensive neuropsychological tests, which are currently used to assess disease severity in RVCL-S.

We aimed to assess the association of serum and CSF NfL and GFAP levels with RVCL-S symptomatology and neuropsychological functioning. We have two important questions: (1) Can NfL and GFAP serve as early marker for pre-symptomatic SVD, and (2) Can NfL and GFAP serve to assess disease progression and cognitive decline.

## Methods

### Subjects

This cross-sectional study was conducted at the Leiden University Medical Center (LUMC), the national referral center for RVCL-S in the Netherlands. (Pre)symptomatic RVCL-S carriers with a proven C-terminal frameshift *TREX1* mutation were included from the LUMC outpatient clinic and the RVCL-ID study [6]. Mutation carriers were classified as symptomatic if they exhibited any of the following: (1) retinopathy; (2) systemic symptoms necessitating treatment; (3) persistent focal neurological deficits; (4) cognitive impairment ranging from mild cognitive impairment to severe/dementia [1, 8]. Non-carrier family members and unrelated individuals were included as controls. Controls were divided in two groups (< 45 years and ≥ 45 years old) to obtain groups with similar age and sex distributions as the mutation carriers.

### Study protocol

Blood samples were obtained from all participants through venipuncture at various times during the day, and participants were not fasting. For participants who provided consent for lumbar puncture, cerebrospinal fluid (CSF) was collected on the same day as the venipuncture. Additionally, the RVCL-ID study participants underwent a brief neuropsychological examination. Three cognitive domains were evaluated through specific test components. Global cognitive functioning was assessed using the Cambridge Cognitive Examination (CAMCOG), which yields a total score ranging from 0 to 107 [22]. Lower scores on this scale indicate poorer cognitive functioning. Psychomotor speed was measured using the Trail Making Test part A (TMT-A) [23, 24]. Participants’ performance was timed in seconds, with longer completion times being indicative for poorer psychomotor speed. Lastly, executive functioning was evaluated using the Trail Making Test part B (TMT-B) [23, 24]. Extended TMT-B times reflect lower executive functioning. Disability was assessed using the modified Rankin scale (mRS) and Barthel Index [25, 26].

### Sample processing

Blood was collected in serum separator tubes and centrifuged at 2000 *g* for 10 min. The supernatant was aliquoted in volumes of 0.5 mL and stored at − 80 °C until analysis. CSF was collected in polypropylene tubes, centrifuged, aliquoted in volume of 0.5 mL each and stored at − 80 °C until analysis. Measurements of NfL and GFAP were performed in duplicate using Single Molecule Array technology (Quanterix, MA USA) with the commercial NF-Light advantage Kit and GFAP Discovery Kit, as previously described [27]. All measurements were performed by certified technicians (Neurochemistry laboratory of the Amsterdam UMC, location VUmc) blinded to clinical information.

### Statistical analysis

Normality of distribution was assessed using histograms and normality plots. NfL and GFAP levels in serum and CSF were non-normally distributed and therefore natural log (ln) transformed to obtain plausible normal distribution for further analyses. The differences between the following groups were investigated: pre-symptomatic RVCL-S mutation carriers versus controls < 45 years, symptomatic RVCL-S mutation carriers versus controls ≥ 45 years, and pre-symptomatic versus symptomatic RVCL-S mutation carriers. For the pairwise comparison of NfL and GFAP levels between groups, multivariate linear regression analysis was performed with adjustment for age and sex. Second, we assessed the correlation between serum and CSF levels of NfL and GFAP using Pearson correlation. Lastly, we performed multivariate linear regression analysis to estimate the correlation between serum NfL and GFAP levels with cognitive functioning (CAMCOG, TMT-A and TMT-B) in RVCL-S carriers. We adjusted for age, sex and education level in all analyses of cognitive functioning. All statistical analyses were performed in SPSS Statistics 25.0 (IBM Corporation, Armonk, NY).

### Standard protocol approvals, registrations, and patient consents

This study was approved by the medical ethics committee of the Leiden University Medical Center. All subjects were ≥ 18 years old and gave written informed consent before participation. The study was performed according to the guidelines of the declaration of Helsinki.

## Results

A total of 108 participants were included: 17 pre-symptomatic RVCL-S mutation carriers (mean age 31.7 years), 22 symptomatic RVCL-S carriers (mean age 55.5 years), 30 controls < 45 years (mean age 33.2 years), and 39 controls ≥ 45 years old (mean age 57.1 years). Serum samples were available from all participants, additionally, CSF samples were collected from 6 pre-symptomatic and 6 symptomatic RVCL-S mutation carriers, as well as from 18 controls < 45 years, and 11 controls ≥ 45 years. Data regarding cognitive functioning was available for 26 individuals with (pre)symptomatic RVCL-S mutations. Table 1 provides a detailed overview of the cohort demographics.

**Table 1:** Demographic and clinical characteristics

	Pre-symptomatic RVCL-S mutation carriers	Symptomatic RVCL-S mutation carriers^a^	Controls < 45 years old	Controls ≥ 45 years old
	n = 17	n = 22	n = 30	n = 39
Age, mean (range)	31.7 (19–52)	55.5 (37–65)	33.2 (22–43)	57.1 (45–73)
Female, n (%)	9 (53)	12 (55)	15 (50)	25 (64)
Hypertension, n (%)	1 (6)	9 (41)	0 (0)	3 (8)
BMI, mean (range)	24.8 (19–32)	25.9 (19–39)	23.8 (18–36)	25.2 (19–33)
Current or past smoking, n (%)	4 (24)	13 (59)	7 (23)	24 (62)
RVCL-S symptoms				
Retinopathy, n (%)	–	18 (82)	–	–
Cognitive complaints, n (%)		2 (9)		
Neurologic complaints, n (%)		7 (32)		
Systemic features, n (%)		7 (32)		
Functioning				
mRS, median (range)	0 (0–2)	1 (0–4)	0 (0–1)	0 (0–1)
Barthel Index, median (range)	20 (20–20)	20 (3–20)	20 (20–20)	20 (19–20)
CSF available, n (%)	6 (35)	6 (27)	18 (60)	11 (28)

*BMI*  body mass index, *CSF*  cerebrospinal fluid, *mRS*  modified Rankin scale, *RVCL-S*  retinal vasculopathy with cerebral leukoencephalopathy and systemic manifestations

^a^Mutation carriers were defined as symptomatic patients when they had (1) retinopathy, and/or (2) systemic symptoms requiring treatment, and/or (3) persistent focal neurological deficits, and/or (4) cognitive impairment ranging from mild to severe/dementia

### Serum and CSF levels of NfL and GFAP in RVCL-S

**Fig. 1:**
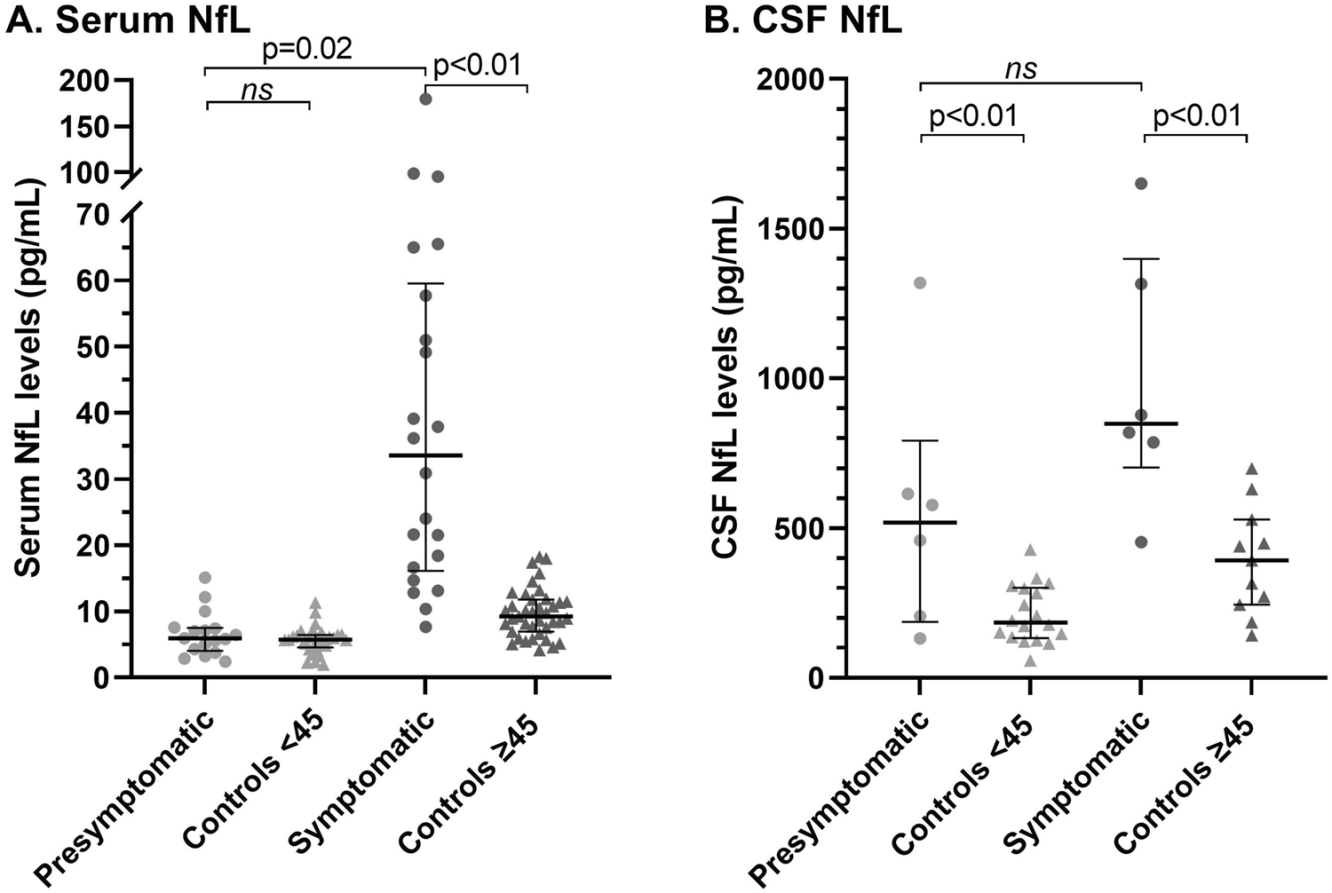
Median (IQR) NfL levels in picogram/milliliter are shown per group in serum (**A**) and CSF (**B**). Symptomatic RVCL-S carriers have increased NfL levels when compared to controls ≥ 45 years in both serum (33.5 pg/mL vs. 9.2 pg/mL, p < 0.01) and CSF (8.5*10^2^ pg/mL vs. 3.9*10^2^ pg/mL, p < 0.01). CSF NfL levels were higher in pre-symptomatic RVCL-S carriers compared to controls < 45 years (5.2*10^2^ pg/mL vs. 1.9*10^2^ pg/mL, p < 0.01). Serum NfL levels were higher in symptomatic RVCL-S carriers compared to pre-symptomatic carriers (33.5 pg/mL vs. 5.9 pg/mL, p = 0.02). All depicted p-values are derived from linear regression analysis of natural log transformed NfL levels and are adjusted for age and sex

CSF NfL levels were higher in pre-symptomatic RVCL-S carriers compared to controls < 45 years (5.2*10^2^ pg/mL vs. 1.9*10^2^ pg/mL, p < 0.01), whereas no differences in serum NfL levels were found between both groups (5.9 pg/mL vs. 5.8 pg/mL) (Table 2, Fig. 1A and B). NfL levels were increased in symptomatic RVCL-S carriers when compared to controls ≥ 45 years in both serum (33.5 pg/mL vs. 9.2 pg/mL, p < 0.01) and CSF (8.5*10^2^ pg/mL vs. 3.9*10^2^ pg/mL, p < 0.01) (Table 2, Fig. 1A and B). Serum NfL levels were higher in symptomatic RVCL-S carriers compared to pre-symptomatic carriers (33.5 pg/mL vs. 5.9 pg/mL, p = 0.02) (Table 2, Fig. 1A).

**Table 2:** Median (IQR) serum and CSF levels of NfL and GFAP

	Pre-symptomatic RVCL-S mutation carriers	Symptomatic RVCL-S mutation carriers	Controls < 45 years old	Controls ≥ 45 years old
	n = 17	n = 22	n = 30	n = 39
Serum NfL (pg/mL) CSF NfL (pg/mL)^a^	5.9 (4.0–7.5) 518 (187–791)	33.5 (16.2–59.5) 847 (702–1399)	5.8 (4.6–6.4) 185 (133–301)	9.2 (6.9–11.8) 392 (244–529)
Serum GFAP (pg/mL) CSF GFAP (pg/mL)^a^	63.0 (48.9–76.9) 9022 (5681–9556)	84.6 (62.8–130.7) 8894 (5619–14,917)	54.3 (41.8–62.0) 6606 (3915–8954)	85.3 (66.0–119.1) 7595 (4901–9289)

*CSF*  cerebrospinal fluid, *GFAP*  glial fibrillary acidic protein, *IQR*  inter quartile range, *NfL*  neurofilament light chain, *RVCL-S*  retinal vasculopathy with cerebral leukoencephalopathy and systemic manifestations

^a^Pre-symptomatic RVCL-S mutation carriers n = 6; symptomatic RVCL-S mutation carriers = 6; controls < 45 n = 18; controls ≥ 45 n = 11

**Fig. 2:**
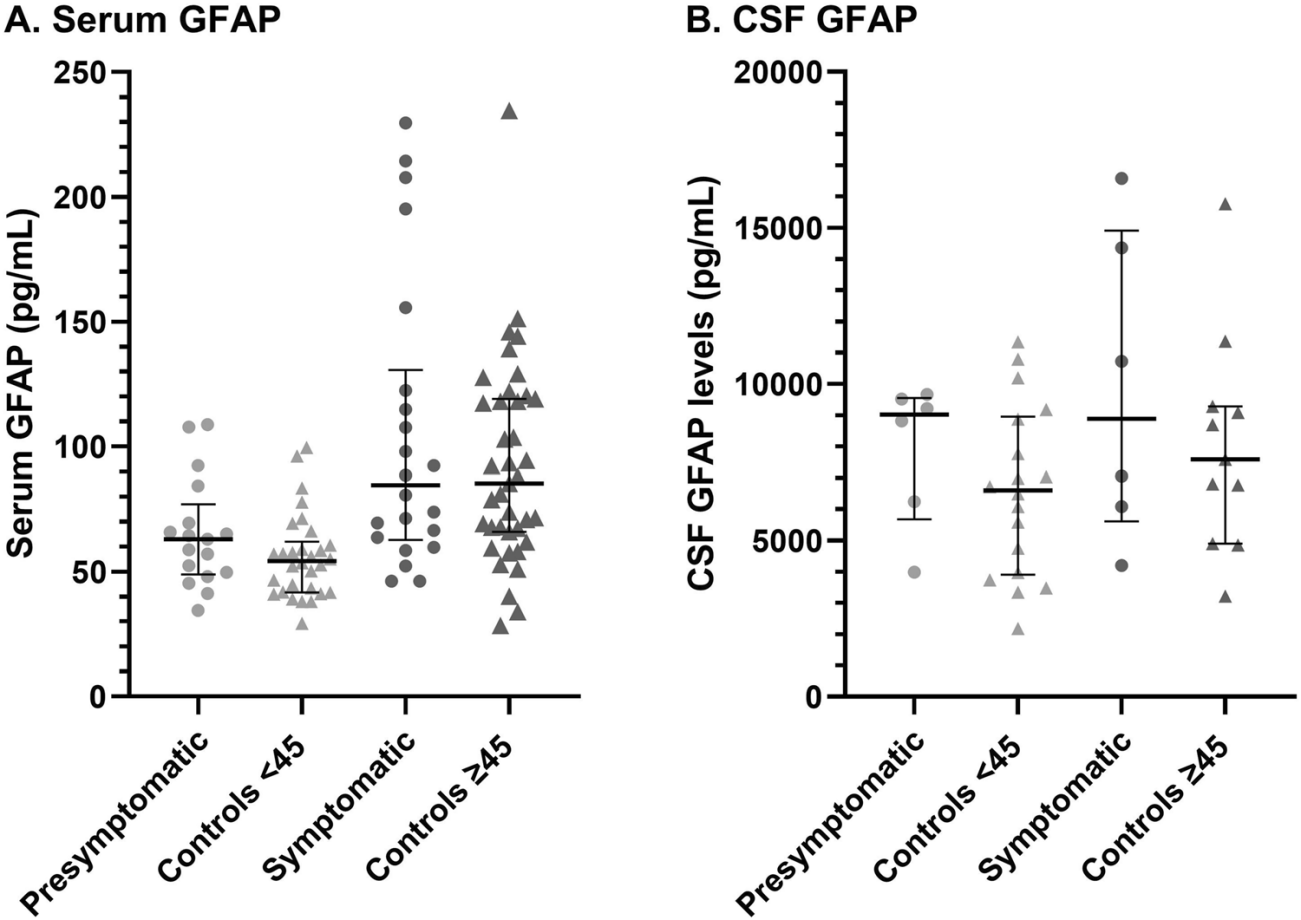
Median (IQR) GFAP levels in picogram/milliliter are shown per group in serum (**A**) and CSF (**B**). No significant differences in GFAP levels were found between groups

Serum and CSF GFAP levels appeared slightly increased in pre-symptomatic RVCL-S carriers when compared to controls < 45 years, however these differences were non-significant (63.0 pg/mL vs. 54.3 pg/mL, p = 0.07, and 9.0*10^3^ pg/mL vs. 6.6*10^3^ pg/mL, p = 0.17, respectively) (Table 2, Fig. 2A and B). Serum GFAP levels were similar between symptomatic RVCL-S carriers and controls ≥ 45 years (84.6 pg/mL vs. 85.3 pg/mL) and CSF GFAP levels appeared slightly increased in symptomatic RVCL-S carriers, but this association was not significant (8.9*10^3^ pg/mL vs. 7.6*10^3^ pg/mL, p = 0.06) (Table 2). No differences in serum and CSF levels of GFAP were found between symptomatic and pre-symptomatic RVCL-S carriers (Table 2, Fig. 2A and B).

All inter- and intra-assay coefficients of variance of NfL and GFAP in serum and CSF were below 10%.

### Correlation between serum and CSF levels of NfL and GFAP

There was a strong correlation between serum and CSF levels of NfL (*r* = 0.76, p < 0.01). Serum and CSF levels of GFAP demonstrated a moderate correlation, (*r* = 0.63, p < 0.01).

### Association between serum NfL and GFAP with cognitive functioning in RVCL-S

**Fig. 3:**
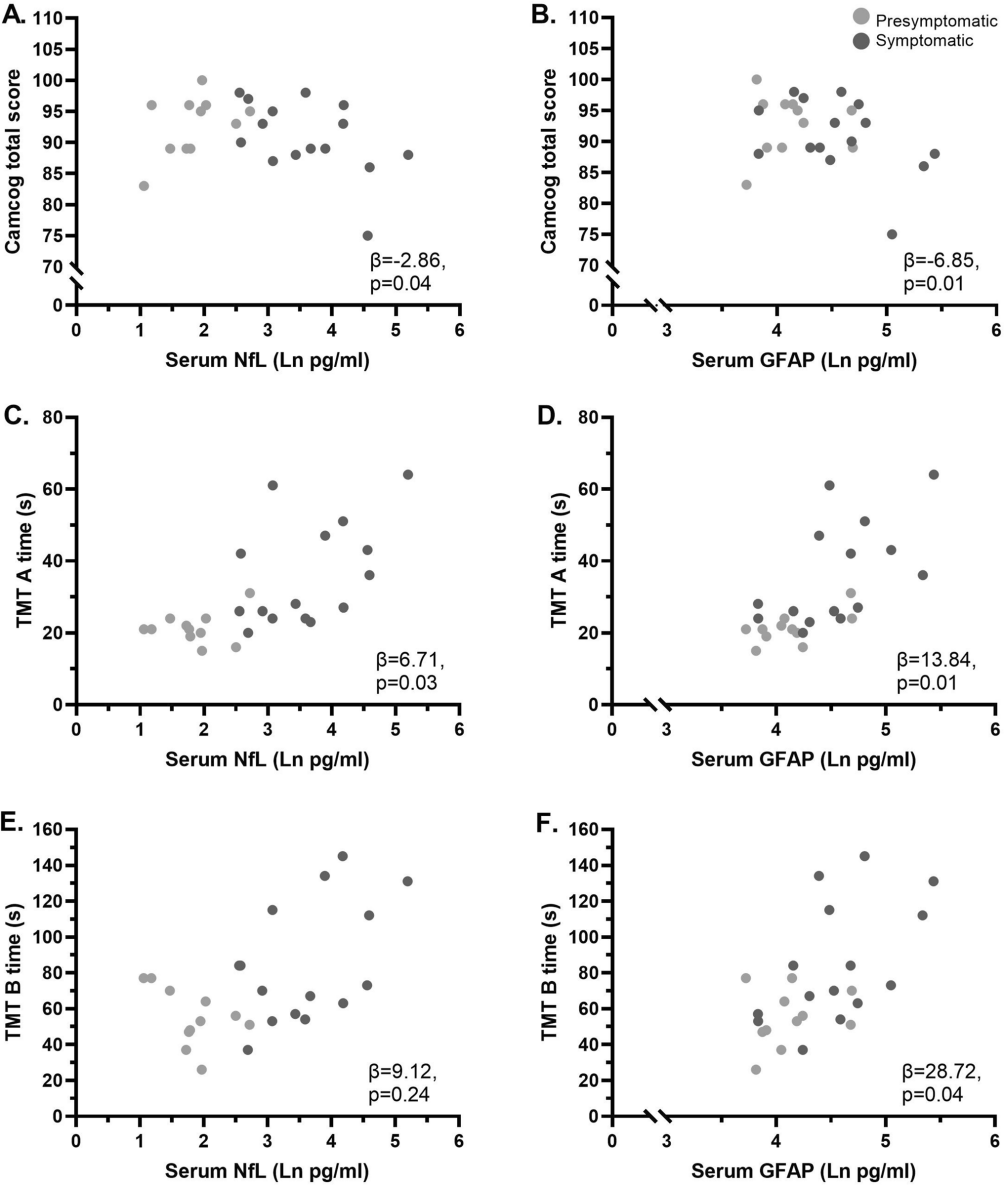
Associations of serum NfL (**A**, **C** and **E**) and GFAP (**B**, **D** and **F**) levels with cognitive test scores in (pre)symptomatic RVCL-S mutation carriers. All depicted regression coefficients and p-values are derived from linear regression analysis of natural log transformed NfL levels and are adjusted for age, sex and education level

In RVCL-S carriers, increasing levels of both serum NfL and serum GFAP were associated with a decrease in the CAMCOG total score (β [95%CI] =  − 2.86 [− 5.58 to − 0.13], p = 0.04 and β [95%CI] =  − 6.85 [− 11.54 to − 2.15], p = 0.01, respectively) (Fig. 3A and B). Increasing levels of both serum NfL and serum GFAP were associated with increasing TMT-A scores (β [95%CI] = 6.71 [0.78–12.65], p = 0.03 and β [95%CI] = 13.84 [3.09–24.60], p = 0.01, respectively), indicating worse cognitive performance (Fig. 3C and D). For TMT-B, higher serum GFAP levels were associated with an increase in TMT-B score (β [95%CI] = 28.72 [1.27–56.18], p = 0.04), indicating worse performance (Fig. 3E and F).

## Discussion

In this cross sectional study in (pre)symptomatic RVCL-S mutation carriers along with controls we examined NfL and GFAP levels to investigate the relationship of these biomarkers with RVCL-S symptomatology and neuropsychological performance. We demonstrate that symptomatic carriers exhibited higher serum NfL levels compared to controls and pre-symptomatic carriers, whereas pre-symptomatic RVCL-S carriers already displayed increased CSF NfL levels compared to controls. While GFAP levels did not significantly differ across groups, elevated serum NfL and GFAP levels in RVCL-S carriers correlated with poorer cognitive functioning, including global cognitive functioning and psychomotor speed. Our study thus indicates that increased serum NfL and GFAP levels can signal impaired cognitive function in RVCL-S carriers, and can potentially serve as markers for SVD progression. Furthermore, CSF NfL levels may offer an early indication of SVD changes, as there is an observed difference in pre-symptomatic RVCL-S patients compared to young controls.

NfL is one of the two core neurofilament proteins in the central nervous system and plays an important role in the growth and stability of axons [28]. Upon neuroaxonal damage or degeneration NfL is released into the CSF and subsequently or in parallel into the blood [14, 28]. Our findings that serum and CSF NfL levels are increased in RVCL-S mutation carriers suggest that neuroaxonal damage plays a role in RVCL-S pathophysiology. This aligns with histopathological examinations conducted on RVCL-S cases, which identified the existence of numerous regions of ischemic necrosis within the white matter. These areas are marked by fibrosis, focal calcifications, reactive astrocytosis, and a concurrent loss of axons [2, 5].

GFAP, a component of the cytoskeleton of astrocytes, is upregulated during reactive astrocytosis, which has been associated with pathological changes in Alzheimer's disease and other neurodegenerative diseases and dementias [19, 21, 29]. Although our study did not reveal significantly higher levels of GFAP in mutation carriers we did find an association between serum GFAP levels and cognitive functioning, similar to a previous study on sporadic SVD, suggesting that GFAP may play a role in RVCL-S disease symptomatology [30].

Our findings on serum NfL and GFAP levels and their association with cognitive functioning in RVCL-S are consistent with previous studies. In both sporadic SVD and CADASIL, serum NfL levels were associated with disease severity and progression, showing its potential as easy accessible biomarker [15–17, 31]. In Alzheimer's disease, serum GFAP levels have demonstrated their predictive value for transitioning from mild cognitive impairment to dementia [19, 32]. Importantly, as NfL is a general marker for neurodegeneration, the increased serum NfL levels found in RVCL-S mutation carriers are quite non-specific. So, while NfL is not suitable as a diagnostic biomarker, the association between serum NfL and cognitive functioning in RVCL-S, shows its promise as a biomarker for monitoring disease symptomatology. However, future studies are warranted to assess the association of NfL and GFAP with radiological, ophthalmological, and systematic signs and symptoms of RVCL-S. Furthermore, longitudinal studies are needed to evaluate the association of NfL and GFAP with disease progression and survival in RVCL-S mutation carriers. These type of studies will potentially lead to biomarkers that can be used to monitor treatment outcomes in future clinical trials.

Our study comes with several limitations. First, the sample size of RVCL-S carriers included for CSF biomarker measurements was small. Nonetheless, there was a robust correlation between serum and CSF concentrations for both NfL and GFAP measurements, aligning well with prior research findings [28]. This is especially promising as venipunctures are less invasive, and repeated measurements more easily acquired. Moreover, a relatively simple serum biomarker clearly has many advantages over more difficult to obtain biomarkers such as high-field MRI markers or extensive neuropsychological tests, which are presently used to evaluate disease severity in RVCL-S. Furthermore, data on cognitive functioning was not available for all mutation carriers. Of note, our group of symptomatic mutation carriers consisted mainly of patients with retinopathy, which is often the first symptom of the disease [6]. Based on previous studies in sporadic SVD and CADASIL one could speculate that serum NfL and GFAP levels will be higher in RVCL-S patients when the disease has further progressed to include neurological and/or cognitive deficits [15, 30, 31]. However, due to the limited sample size no differentiation between ophthalmological, cerebral and systemic manifestations of the disease could be made. Nevertheless, the demonstration of NfL and GFAP's potential as biomarkers for cognitive function in RVCL-S validates the promising direction of this research. While we were not able to match our cohort on age and sex, we did obtain groups with a similar age and sex distribution. To ensure that no residual confounding occurred we corrected for age and sex in all our analyses. Next, as this was an exploratory study we did not correct for multiple testing. Finally, due to the cross-sectional nature of the study design, we were unable to evaluate how NfL and GFAP are linked to RVCL-S disease progression and survival in RVCL-S mutation carriers. A strength of our study is the ultra‐sensitive single‐molecule array (Simoa) for measurements [27]. Most importantly, we present a hereditary neurovascular model for SVD and vascular dementia, which provides the opportunity to evaluate the pre-symptomatic stage of SVD.

In conclusion, serum and CSF NfL levels are increased in symptomatic RVCL-S patients and CSF NfL levels are already increased in pre-symptomatic mutation carriers. Moreover, both serum NfL and serum GFAP levels are associated with cognitive functioning in RVCL-S, showing the potential of these neuronal injury and astrogliosis biomarkers for monitoring disease progression and cognitive decline for SVD.

## Data Availability

Anonymized data used for this study are available from the corresponding author for the purpose of research only, upon reasonable request.

## Abbreviations

CADASIL: Cerebral autosomal dominant arteriopathy with subcortical infarcts and leukoencephalopathy
CAMCOG: Cambridge cognitive examination
CSF: Cerebrospinal fluid
GFAP: Glial fibrillary acidic protein
NfL: Neurofilament light chain
RVCL-S: Retinal vasculopathy with cerebral leukoencephalopathy and systemic manifestations
SVD: Cerebral small vessel disease
TMT-A: Trail Making Test part A
TMT-B: Trail Making Test part B
